# Supplementary material for: Novel Driver Strength Index highlights important cancer genes in TCGA PanCanAtlas patients
Source: PeerJ. 2022 Aug 11;10:e13860. doi: 10.7717/peerj.13860 (PMC9375969; doi:10.7717/peerj.13860)
Supplement: Supplemental Information 2 [file peerj-10-13860-s002.zip › COHORTS/genes plots/age/2021_8_16_14_9_distribution_cohorts_40-44.pdf]

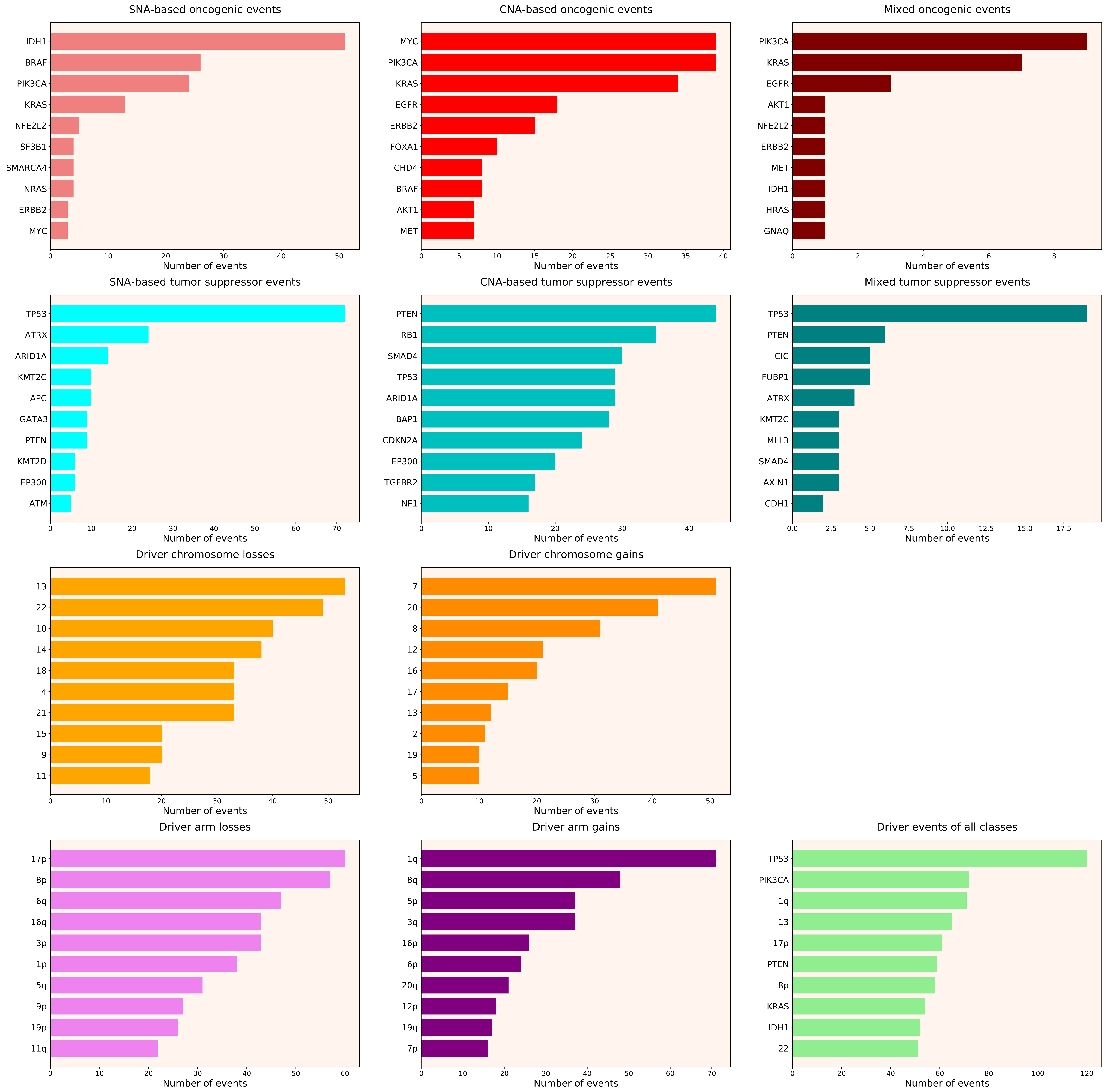
SNA-based oncogenic events
IDH1
BRAF
PIK3CA
KRAS
NFE2L2
SF3B1
SMARCA4
NRAS
ERBB2
MYC
Number of events
CNA-based oncogenic events
MYC
PIK3CA
KRAS
EGFR
ERBB2
FOXA1
CHD4
BRAF
AKT1
MET
Number of events
Mixed oncogenic events
PIK3CA
KRAS
EGFR
AKT1
NFE2L2
ERBB2
MET
IDH1
HRAS
GNAQ
Number of events
SNA-based tumor suppressor events
TP53
ATRX
ARID1A
KMT2C
APC
GATA3
PTEN
KMT2D
EP300
ATM
Number of events
CNA-based tumor suppressor events
PTEN
RB1
SMAD4
TP53
ARID1A
BAP1
CDKN2A
EP300
TGFBR2
NF1
Number of events
Mixed tumor suppressor events
TP53
PTEN
CIC
FUBP1
ATRX
KMT2C
MLL3
SMAD4
AXIN1
CDH1
Number of events
Driver chromosome losses
13
22
10
14
18
4
21
15
9
11
Number of events
Driver chromosome gains
7
20
8
12
16
17
13
2
19
5
Number of events
Driver arm losses
17p
8p
6q
16q
3p
1p
5q
9p
19p
11q
Number of events
Driver arm gains
1q
8q
5p
3q
16p
6p
20q
12p
19q
7p
Number of events
Driver events of all classes
TP53
PIK3CA
1q
13
17p
PTEN
8p
KRAS
IDH1
22
Number of events
